# Selective spatial attention modulates bottom-up informational masking of speech

**DOI:** 10.1038/srep08662

**Published:** 2015-03-02

**Authors:** Simon Carlile, Caitlin Corkhill

**Affiliations:** 1 School of Medical Sciences and The Bosch Institute, University of Sydney, Sydney, NSW 2006, Australia; 2 School of Medical Sciences, University of Sydney, Sydney, NSW 2006 Australia

## Abstract

To hear out a conversation against other talkers listeners overcome energetic and informational masking. Largely attributed to top-down processes, information masking has also been demonstrated using unintelligible speech and amplitude-modulated maskers suggesting bottom-up processes. We examined the role of speech-like amplitude modulations in information masking using a spatial masking release paradigm. Separating a target talker from two masker talkers produced a 20 dB improvement in speech reception threshold; 40% of which was attributed to a release from informational masking. When across frequency temporal modulations in the masker talkers are decorrelated the speech is unintelligible, although the within frequency modulation characteristics remains identical. Used as a masker as above, the information masking accounted for 37% of the spatial unmasking seen with this masker. This unintelligible and highly differentiable masker is unlikely to involve top-down processes. These data provides strong evidence of bottom-up masking involving speech-like, within-frequency modulations and that this, presumably low level process, can be modulated by selective spatial attention.

The intelligibility of a talker of interest against a background of concurrent talkers is degraded as a consequence of masking (the cocktail party problem; for recent review see Ref. 1). The spectral overlap between the target talker and the maskers will result in energetic masking when the non-target energy is dominant. Research over the last few decades has demonstrated that other aspects of the competing talkers also contribute substantially to the masking and this is generally referred to as informational masking (see Ref. 2 for review). Although originally a classification by exclusion (i.e. not energetic masking), more recent work indicates that informational masking may result from a number of different processes.

Similarity between talkers is a particularly strong driver of informational masking. Often errors in identifying the target sentence represent words spoken by the masker talkers^3^,^4^. This indicates that audibility is not the problem; rather it is the confusion between talker streams and the misattribution of words to talkers. It is likely that knowledge based schema such as semantic context and prosody play a role in helping to segregate multiple concurrent talkers and manage such talker confusion errors (e.g. Refs. 5, 6). Indeed, familiarity with the target talker^7^,^8^, knowing where^9^ or when^10^ to listen and virtually any perceived physical difference such as spatial location^11^,^12^ or voice quality^3^ all play important roles in reducing informational masking. All of these findings indicate a role for attention in the processes of successfully parsing the different streams of concurrent speech and sustaining selection of the appropriate stream for the task at hand (see for e.g. Ref. 13). Information masking could result from a failure of these top-down processes, either as a result of processing load or ambiguity in the auditory scene which undermines segregation and streaming (e.g. Refs. 14, 15).

Cueing “what/who” or “where” provides the opportunity for the listener to steer auditory attention so as to enhance the formation of auditory objects and streams and to select the appropriate target stream. Attention of course, is a process of biased competition involving top-down, endogenous attention and bottom-up or exogenous attention driven by salience in a stimulus (see for review Ref. 16 and commentary^13^). One form of informational masking could rely on exogenous attention, elicited by a particularly salient stimulus, drawing attention to an object or stream that is not appropriate to the task. This could be the basis of the so-called “odd-sex” distractor phenomenon^3^ where including say a female talker masker with a male target and another male masker talker produces more informational masking that would have occurred had all the talkers been of the same gender. This would be an example of stimulus driven or bottom-up masking.

There are hints in the literature of another type of bottom-up informational masking. Brungart and colleagues^17^,^18^ used an across-ear interference task to examine informational masking produced by a number of synthetic speech stimuli: Recognition of a target sentence presented over headphones with a concurrent masker sentence in the same ear was strongly modulated by a masker presented to the contralateral ear. Broadband speech stimuli produce strong interference while spectrally matched noise produced none. The effectiveness of synthetic, modulated noise band speech and sine wave speech as interferers was related to the intelligibility of the speech which depended on the number of frequency bands used to construct the speech. Surprisingly, speech constructed using only one or two frequency bands still produced some interference even though intelligibility was low. This suggests that intelligibility *per se* is not necessary to produce interference. These authors conclude that the speech-like, amplitude modulation in the contralateral masker stimuli interferes with some “preattentive central auditory processing mechanism” – presumably a bottom-up process. Gallun et al^19^ also report that the effectiveness of an across ear masker was also dependent on the temporal-spectral similarity to the target.

There is also evidence that other non-intelligible sounds can also exert informational masking such as time reversed speech^20^ or unintelligible foreign speech (Refs. 20, 21 but see also Ref. 12). The masking produced by these stimuli could also be, to some extent, the result of exogenous deflection of attention from the discrimination task as both types of maskers are voiced (albeit unintelligible) speech. Chen and colleagues^22^ used harmonic complexes and manipulated the F0 contours as well as segmented the complexes with speech shaped noise to produce non-intelligible, speech-like stimuli but with the absence of voiced qualities. In that study, perceived differences in the locations of the target and maskers suggested informational masking of 2 dB to 3 dB depending on the stimulus. Amplitude modulation in non-speech stimuli are also believed to play a role in masking speech over and above the energetic masking of such stimuli (e.g. Refs. 23, 24) and more recently might even explain much of the masking seen with steady-state broadband maskers^25^.

The processes leading to unmasking can be highly dynamic. Listeners can take advantage of the rapid amplitude modulations in the maskers to glimpse elements of the target speech^26^,^27^,^28^ although this is probably restricted to reducing energetic masking^29^. Even when a significant proportion of the target words are inaudible due to energetic masking, the auditory system is able to perceptually fill in the missing information – so called phonemic restoration^30^. This most likely represents processing at a range of levels including spectro-temporal induction, as well as reflecting lexical, linguistic and semantic expectations about the content of meaningful speech (e.g. Refs. 31, 32). In this context, one surprising result is that when intervening noise is amplitude modulated by the amplitude of the missing speech, intelligibility is increased^33^ suggesting that some useful speech information is contained in the gross amplitude envelope. Notwithstanding the linguistic contributions to phonemic restoration, the fast time course of these processes is consistent with automatic, bottom-up processes contributing to the release from masking.

In this study we were interested in examining the extent to which informational masking in a speech-on-speech masker intelligibility task can be accounted for by bottom-up sensory processes rather than a failure in top-down attentional or other cognitive processes. To that end we have produced an unintelligible speech-like stimulus (‘garbled’ speech) where the within-channel modulations are identical to intelligible speech. To preview our result - using a spatial release from masking paradigm we have found that a substantial proportion of the informational masking produced by the speech masker can be accounted for by the unintelligible speech-like masker. This suggests that the within-channel masker modulation plays a key role in bottom-up informational masking but can also be modulated by spatial attention when selecting the target stream based on location.

## Results

**Figure 1:**
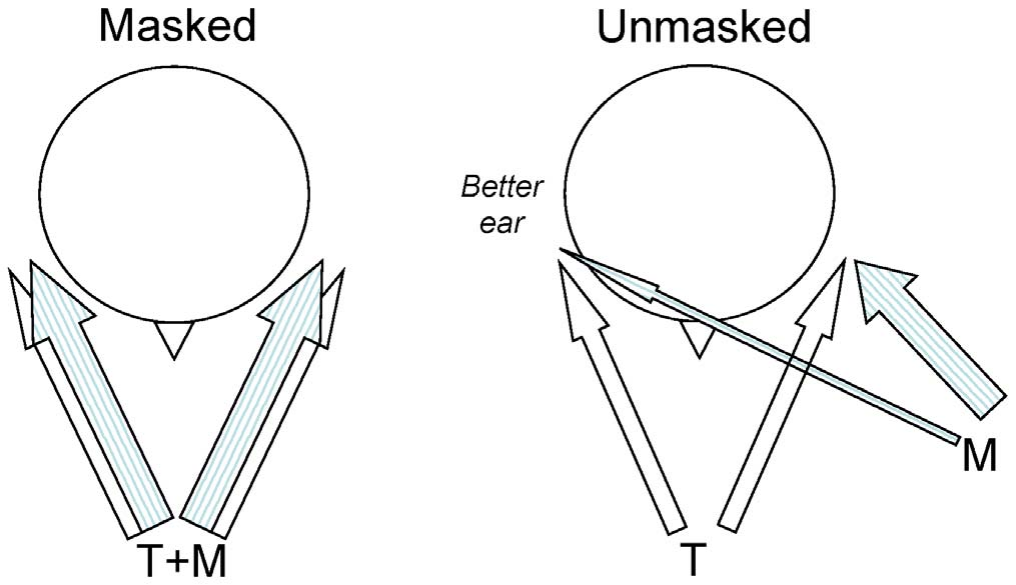
The virtual auditory space (VAS) listening paradigm is illustrated showing the relative placement of the targets (T) and maskers (M) in the masked (collocated) and unmasked (separated) conditions. The “better ear” is identified in terms of the target to masker ratio at the ear furthest from the masker in the unmasked or separated condition.

The principal aims of this experiment were to test the capacity of the temporally “garbled” speech masker to produce informational masking compared to normal speech and speech-matched modulated noise and to determine if spatial selective attention could modulate that masking. The speech reception thresholds (SRT) were measured by varying the level of the target sentence in a constant masker background and defined as the target to masker ratio producing 50% correct target word identification. The SRT with the target collocated with each masker provide a measure of the total masking (energetic and informational) produced by the different maskers. Moving the maskers to one side (60° to the left; Figure 1) resulted in an improvement in the SRT and is referred to as the total spatial release from masking. Part of this improvement in SRT will result from the reduction in the masker level in the ear furthest from the masker – so called “better ear” listening (Figure 1; e.g. Refs. 34,35,36,37) and provide an estimate of the release from energetic masking: i.e. improvements that simply reflect the energetic improvement in the target to masker ratio in the better ear. Differences between the “better ear” masking release and the total spatial release from masking will largely reflect informational masking release (but see also Ref. 38). If the within channel modulation characteristics of the garbled masker are playing an informational masking role as predicted from the above, then there should be a significant difference between the total spatial release from masking and the SRT at the better ear. Importantly, comparison of the magnitude of this difference with that for the speech masker will provide a measure of the relative contribution of this bottom-up component of informational masking.

To measure these separate contributions we presented the stimuli over headphones in virtual auditory space (VAS see Methods). Presenting the “better ear” masker stimulus diotically for the separated condition maintains this energetic masker advantage which can then be estimated by measuring the SRT. Critically, this approach eliminates the perception of differences in the locations of the target and maskers^35^, a perception that facilitates release from informational masking (e.g. Ref. 11).

In summary, four stimulus conditions were used. The SRTs were measured for target and maskers presented in VAS (i) collocated in front and (ii) with the maskers 60° to the right (Figure 1). Using the better ear signal, stimuli were also presented diotically (identical in both ears) for both the (iii) collocated and (iv) the separated conditions. Conditions (i) and (iii) should (and did) produce identical SRTs because the sound levels in each ears will be the same for locations in front of the listener. The difference between the SRTs for (i) and (ii) provides an estimate of total spatial release from masking and the difference between (iii) and (iv) will provide an estimate of the energetic masking release. The difference between the total spatial release from masking and the release from energetic masking can be largely attributed to the release from informational masking.

**Figure 2:**
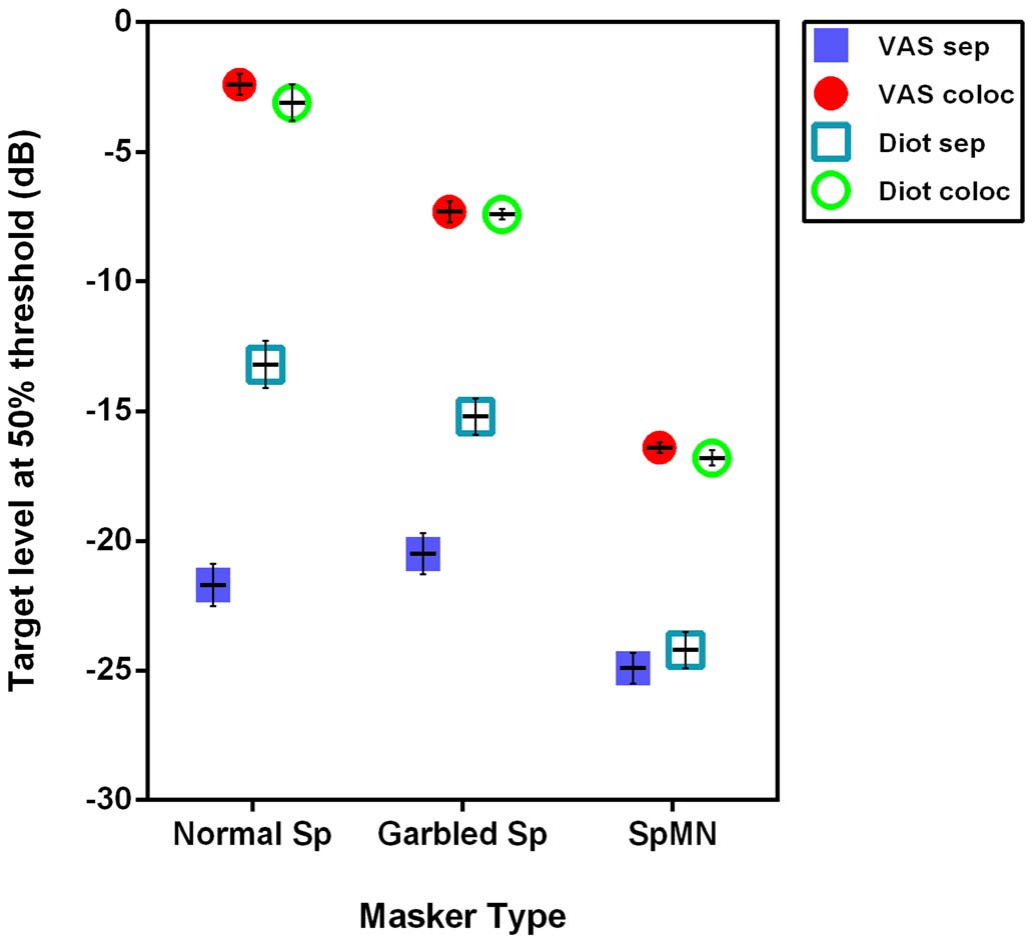
The mean speech reception thresholds are plotted for each masker and listening condition Error bars indicate standard errors of the mean. The data for different listening conditions: VAS collocated- red filled circles; VAS separated – blue filled squares; diotic collocated – green open circles; diotic separated (or “better ear” stimulus) – light blue open squares.

The mean SRT calculated for the group of 8 subjects are plotted for each of the different maskers (Figure 2). Given that each masker type varies significantly as an informational masker we would expect differences in the SRT for the collocated condition (Figure 2: red filled circles). The least effective masker was the speech-matched noise (SRT −16.4 dB) while the most effective masker was the normal speech masker (SRT −2.4 dB) with the garbled speech masker producing an SRT (−7.3 dB) closer to that of the speech masker.

There was a very good correspondence between the SRTs obtained for the VAS collocated and the left ear diotic collocated conditions for each listening condition (Figure 2: red filled circles and green open circles). This is consistent with the assumption that, for locations on the midline, the input to the two ears should be effectively the same. What minor interaural differences that have been reported for anterior midline locations^39^ do not seem to be contributing significantly to this task. In addition, this finding suggests that the perception of externalisation present in the VAS condition but absent in the diotic condition did not contribute to the unmasking when the stimuli were collocated.

When the target and maskers are separated, thresholds are thought to approach a listener's best performance because of the release of both EM and IM (see for instance Ref. 40). Consistent with this, the range of SRTs for the VAS separated condition (Figure 2: dark blue filled squares) was much smaller (around 3 dB) when compared with the collocated condition (filled red circles: range around 15 dB): In particular the SRTs for the normal speech and garbled speech maskers were −21.7 dB and −20.5 dB respectively while the speech matched noise was the lowest of any condition at −24.9 dB.

As discussed above the SRTs obtained for the diotic separated condition for each masker (Figure 2 open light blue squares) provides a measure of the contribution of better ear listening and an estimate of the release from energetic masking. For the noise masker, there was almost no difference between the separated and diotic separated conditions (Figure 2, dark blue filled square c.f. light blue open square) indicating that the total spatial release from masking with the noise masker can be explained almost entirely by the change in the energetic masking afforded by “better ear” listening (see also below). In contrast, the SRT for the speech masker in the spatially separated condition (SRT −21.7 dB; dark blue filled square) was much lower than for the diotic separated condition (SRT −13.2 dB; light blue open square). This indicates a substantial release in non-energetic or informational masking. Most interestingly, and the key finding in this study, was that for the garbled masker there was also a large difference between the VAS separated SRT (−20.5 dB; dark blue filled square) and that for the diotic separated condition (−15.2 dB; light blue open square). This indicated a substantial release of non-energetic or informational masking when the garbled masker was spatially separated from the target talker.

**Figure 3:**
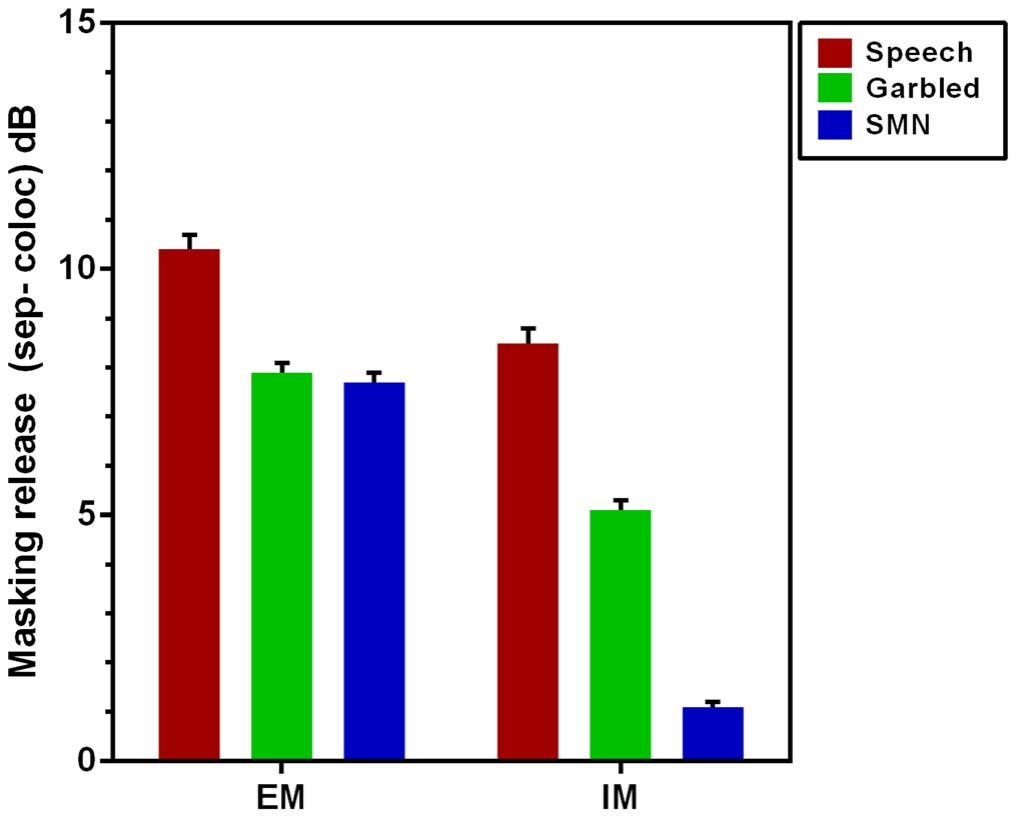
The mean spatial release from masking calculated from the data in Figure 2 is plotted for each masker (±1 SEM). The estimate of the energetic masking release was obtained from the difference between the collocated SRTs and the better ear SRTs for each subject. The estimate of the informational masking is obtained from the differences between the total spatial release from masking and better ear SRT for each subject.

The difference between the VAS collocated and the VAS separated thresholds provides a measure of the total spatial release from masking. The “better ear” diotic thresholds (Figure 2, light blue squares) compared to the collocated thresholds (red filled circles) provides an estimate of the energetic masking release, which when subtracted from the total masking release (Figure 2, dark blue squares) then provides an estimate of the non-energetic or informational component. The relative components of the energetic and informational masking release are plotted in Figure 3.

All maskers demonstrate a significant energetic masking release when the target and maskers are separated (Left bars Figure 3; 8 to 10 dB; p < 0.5 as 1.96 × SEM do not overlap 0 dB). As expected the speech maskers demonstrate a significant fraction of non-energetic or informational masking release (8.6 dB) while the garbled speech maskers demonstrate more than half that (5.2 dB). Interestingly, the speech shaped modulated noise masker showed a small but significant 1.1 dB information masking release (p < 0.05 as 1.96 × SEM do not overlap 0 dB).

## Discussion

The principal finding of this study was that, using a spatial unmasking paradigm, the unintelligible speech-like masker demonstrated significant and substantial non-energetic masking. Before considering the potential basis of that non-energetic masking and its release, it is useful to consider the spatial unmasking demonstrated by the speech masker and the speech matched noise – both of which have been used previously in studies of energetic and informational masking.

In comparing the masking produced by the speech maskers, Brungart and colleagues^3^ used substantially the same corpus and tested the masked thresholds using a diotic presentation similar to that for the “better ear” conditions in this study. They report masked thresholds for the speech maskers of around −1 dB SNR (their Figure 2) which compares well with the grouped mean of −3.1 dB for the diotic collocated and the −2.4 dB for the VAS collocated found here. Three different effects may have contributed to small differences between these studies. Firstly, subtle difference between the listening groups: The listeners in this study were acclimatised to Australian English and the CRM corpus is recorded with North American accents. Accent differences between listeners and talkers, however, might have been expected to have made the task harder i.e. produced more positive SNRs. Secondly, the version of the corpus used here was broadband out to 16 kHz. This was chosen to maximise the localisation cues available to support the spatial unmasking (see Ref. 41). The previous study^3^ used a version low passed at 8 kHz so the additional spectral information available in this study may have provided a small benefit for discriminating the target talkers from the maskers. Thirdly, in this study both the target and masker talkers had been filtered with the individuals HRTFs for presentation in VAS whilst in the previous study the speech was not filtered before headphone presentation. The broad conchal gain around 4 kHz and the variable mid frequency notch (~6 kHz to 8 kHz) in the HRFT filtering may have acted to change the within frequency band intelligibility weights and changed the relative importance of different frequency ranges in determining intelligibility (see Ref. 42). Hawley and colleagues measured SRTs using non-individualised VAS^34^ (their figure 2) and reported a threshold 3 dB target to masker ratio. This corresponds to an SRT of around 0 dB based on overall signal to noise. In this case the slightly higher value probably reflects the use of same talker for target and maskers which has a higher level of informational masking (around 3 dB for the CRM corpus^3^) making their finding directly comparable with that found here.

The total release from masking in the VAS separated condition for the two talker speech masker reported here was 19 dB, somewhat higher than that seen previously for one talker masker presented under similar free field condition (13.7 dB^11^, their figure 4, F-R condition). This is consistent with the finding that two talker maskers are likely to exerting somewhat more informational masking^3^ which is then released when the target and maskers are in different locations. The estimate of the release from non-energetic masking of 8.6 dB obtained by subtracting the “better-ear” SRT is in good agreement with the informational masking release seen for two talker masker of around 10 dB when energetic masking is controlled for (see Ref. 12, their figure 3(b)), particularly given the differences in the methodology and the speech materials used.

In comparing the masking produced by the speech matched noise, the speech matched noise used in this study was similar to the noise used by Brungart and colleagues^3^ so it was somewhat surprising to find that in this study the noise was around 5 dB less effective as a masker in the VAS and diotic collocated conditions (−11 dB Brungart et al^3^ c.f. −16.4 dB and −16.8 dB respectively found here). Subsequent examination of the level spectrum of these stimuli revealed that, when compared to the long term average of the speech target and the speech and garbled maskers, the noise in this study was 6 to 7 dB below those levels over the frequency range 1.2 kHz to 3.5 kHz. The articulation index substantially weights these frequencies indicting a significant contribution to speech intelligibility^42^ and may well explain the reduced capacity of this energetic masker compared to the previous study^3^. This difference in level may have resulted from the relatively small number of tokens used to estimate the long term spectrum of the corpus in our study (54 sentences) compared to the 2048 sentences used by Brungart and colleagues^3^. Nonetheless, the principal reason for using a noise masker was to verify the assumptions behind a “better ear” estimate of energetic masking. In that regard, the very close correspondence between the SRTs for the diotic separated (24.2 dB) and the VAS separated (−24.9 dB) for the SMN confirms this expectation. The small IM for this stimulus (Figure 3, blue bar) is also consistent with that reported by Marrone et al^43^ for symmetrically placed noise maskers, which largely eliminated any better ear effect and may represent a binaural processing advantage (see also Ref. 38).

Having established the correspondence between the data here and previous studies for the speech and speech matched noise maskers we can now turn our attention to the effects of the garbled masker. Separating the garbled masker from the target talker produces a substantial and significant unmasking (13.2 dB) of which 7.8 dB could be attributed to energetic masking. The remaining fraction (5.4 dB) represents a substantial unmasking –larger than that reported for symmetrically placed time reversed speech maskers^40^ and double that reported by Chen et al^22^ using their speech like unintelligible maskers. There are a number of possible processes underlying this release from masking.

One classic view of informational masking with speech maskers is that the masker words are misattributed to the target stream (e.g. Refs. 3, 4). This becomes less likely with increased differences between the masker and the target such as differences in spatial location of the sources (e.g. Refs. 11, 12) or differences in the quality of the voices (e.g. Ref. 3). In the case of the garbled masker, the unmasking can be attributed to differences in the location of the sources, however, as the perceived quality of the garbled speech is very different to the quality of the target voice it is very unlikely that portions of the garbled masker were attributed to the target talker. In any case, as the garbled masker was unintelligible, it would not be possible for masker words to be misattributed to the target steam.

A second means by which a masker might decrease performance on a speech recognition task is by triggering exogenous attentional shifts to the masker stream thus diverting processing from the target stream. With time reversed speech (e.g. Ref. 40) and unintelligible foreign language speech (e.g. Ref. 20), the voiced quality of the talker and the natural amplitude variations in the speech could both act to trigger exogenous attention shifts and account for some of the IM masking reported previously. While we cannot discount this possibility in explaining these results we do not favour this explanation. Firstly, the garbled masker is relatively homogenous in its level and content: In normal speech the temporal correlation across frequency bands means that the overall amplitude envelope of the speech changes substantially from moment to moment thus allowing for level driven changes in saliency. By contrast, the across band decorrelation in the garbled masker ensures a more even level over time. This also largely eliminates bursts of broadly distributed high frequency energy associated with the fricatives and plosives. Secondly, the overall levels of the maskers in the collocated and separated conditions are not that much different from the point of view of level driven saliency so spatial separation is also not likely to modulate endogenous attention shifts. As the garbled masker is entirely unintelligible (even more so than multi-talker babble) it is difficult to see what other aspects of the content might drive changes in saliency.

A third possibility is that the garbled speech masker is exerting a form of informational masking that is not reliant on the top-down processes discussed above. The ability to form auditory objects, to stream speech from a particular source and to understand the speech will be dependent on the fidelity of the encoding of the information (see Refs. 13, 44, 45, 46). A notable characteristic of the garbled masker is that the within channel content of the masker is identical to the speech masker. Speech is a highly redundant signal and it has been known for some time that speech generated using amplitude modulation within a relatively small number of frequency bands is sufficient to produce highly intelligible speech (e.g. Refs. 47, 48). These data suggest that the speech like amplitude modulations in the garbled masker is producing interference in the bottom-up processing within the relevant frequency bands. Such low level interference would reduce the fidelity of the processed signal by interfering with the within-band encoding of extracted modulation information. This is also consistent with the observation that spectral-temporal similarities between target and maskers increase across-ear interferences and Gallun et al^19^ discuss this specifically in the context of the degradation of the grouped auditory object. When the magnitude of the IM produced by the garbled speech masker (5.4 dB) is compared with the matched speech maskers (8.6 dB) this suggests that a very substantial fraction of the IM seen with speech on speech masking could be attributed to bottom-up interference rather than a failure of top-down attention.

Such an interpretation is consistent with recent work examining the role of within-channel modulation from notionally steady state noise in masking concurrent speech. Stone and colleagues^23^,^24^,^25^ have provided strong evidence that speech masked by broadband noise is more likely the results of modulation interference or masking produced by the fluctuations at the output of each auditory channel. Such modulations result from the intermodulation of the different spectral components within each channel and has been referred to as a “form of informational masking^24^” that is clearly of the bottom-up variety. The large IM produced here by the garbled masker demonstrates the importance of this effect when all the relevant modulation channels are affected simultaneously. Another factor potentially contributing to the magnitude of the effect with the garbled masker is the actual shape of the modulation envelope. Traditionally, the use of sinusoidally modulating maskers (so-called SAM stimuli) in studies of modulation interference is based on the assumption of temporally symmetrical modulation envelopes; a condition that is probably not strictly the case for within channel speech modulations but would be largely preserved in the garbled maskers produced here.

Another key finding is that the within-channel modulation interference appears to be modulated by the application of spatial attention or at least by the spatial separation of the speech target and the garbled maskers. Spatial separation is thought to aid stream selection and support continuity – a post grouping phenomena (see Refs. 13, 40) - while spatial cues are thought to provide only weak cues for grouping (for review Ref. 49). Presumably, the modulation masking produced by the garbled maskers would be at an early, within-channel level of processing. What is surprising then, is that the focus of spatial attention, which underlies to a large extent the spatial release from information masking, is also able to modulate this bottom-up form of masking.

Recent work has demonstrated that auditory cortex contains a detailed acoustic representation of the phonemes of speech^50^,^51^. Within-channel signals that share some of the acoustic characteristics of natural speech are likely to interfere with such feature detectors and degrade or mask the encoding of features associated with the target talker. Of considerable interest, however, is the finding here that spatial separation of the garbled masker from the target talker produces an increased unmasking over and above the SNR changes produced at the better ear. This suggests that, despite any low-level interference, other perceptual differences in the sources (in this case location) could be leveraged, presumably by the application of focussed spatial attention, to enhance the processing of the target. Evidence is accumulating from multi-electrode array recordings from human auditory cortex (e.g. Refs. 50, 52, 53) and MEG recordings (e.g. Ref. 54) that early auditory cortical representations reflect the acoustic properties of the collection of concurrent signals but that these representations already appear to demonstrate some object-like invariance and can be modulated by top-down attention. In higher order auditory cortex the encoding appears to become more selective for the “attended to” object^55^. While the data here do not speak to any potential locus of interaction between the garbled masker and attended to speech target, these new electrophysiological data provide tantalising hints as to where forms of bottom-up informational masking could arise. Whether the application of attention modulates the early cortical representations or exploits the significant efferent auditory pathway to modulate bottom masking further up-stream is a related question of some significance.

## Methods

A total of eight subjects participated in this experiment (4 female and 4 male; ages 19–26 years, mean 21.3 years). All had normal hearing by self-report. All experiments were carried out in accordance with the approved guidelines and were approved by the Human Ethics Research Committee of the University of Sydney and all subjects provided written informed consent.

### Test stimuli

All target and masker stimuli were either samples from a broadband version (80 Hz to 16 kHz) of the Coordinate Response Measure (CRM) Corpus^56^ or were derived from such samples. The CRM Corpus consists of 8 sets of 256 individually recorded sentences, each set spoken by a different talker (4 male and 4 female). All sentences in the corpus are of the same structure; “ready <callsign> go to <colour> <number> now”, with all combinations of 8 call signs (arrow, baron, charlie, eagle, hopper, laker, ringo or tiger), 4 colours (blue, green, red or white) and 8 numbers (1–8) in each set. Subjects were required to respond to target stimuli which were identified by the call sign “baron” by entering the appropriate colour and number on a small touch screen. Responses were judged correct only when both the selected colour and number matched those spoken by the target voice. Unless otherwise stated, the sentences were band passed at 80 Hz to 16 kHz.

### Masker stimuli

There were three types of masker stimuli. Normal speech maskers were comprised of two female talkers drawn randomly from the CRM corpus. The two talkers were combined so that the fixed overall RMS level matched the target talker at SNR 0 dB. The talkers were always different from the target talker and used a call sign other than “baron”.

To generate the “garbled” speech masker, the female talker masker sentences were passed through a 22 band filter bank (Matlab V6, The Mathworks, filterdesign toolbox – order 10 k) spaced on an ERB scale from 50 to 16.5 kHz^57^ (see table 1 for cut off frequencies). Each masker sentence was between 1.64 and 2.45 seconds in length (mean: 1.91 s; standard deviation: 0.15 s) and the output of each filter was treated as a circular buffer. The sentences were reconstructed by randomly selecting a start point within the buffer for each filter and then summing the signals from all of the different frequency bands for output. In this way, the within frequency band, speech-like characteristics of each original masker sentence was preserved and the stimulus sounded similar to speech babble but was completely unintelligible. The two randomly selected garbled female masker sentences were then combined as above to provide the garbled speech masker.

A purely energetic noise masker (speech-matched noise) was also generated and matched to the temporal and spectral characteristics of the female masker talkers. The long term spectral average of the masker talkers was obtained for 56 masker talker samples (14 from each female talker in the corpus) using Welch's method of spectral estimation (Matlab, pwelch). An FIR filter was derived from the measured spectrum and convolved with 30 seconds of Gaussian noise. A library of speech-matched noise stimuli was generated by randomly selecting segments of the noise to match the duration of each pair of masker talkers. So as to provide amplitude modulations similar to speech and, therefore the opportunity for glimpsing the target^27^, the overall amplitude envelope of the masker talkers was obtained for each masker talker by rectifying the output of a second order Butterworth low-pass filter and was applied to the segment of speech spectrum noise. Pairs of speech matched noise maskers were selected randomly and combined as above. This stimulus was unintelligible.

### Generation of individualized virtual auditory space

Stimuli were presented in individualised virtual auditory space (VAS^58^). Virtual auditory space was generated by filtering sounds presented over headphones using the acoustic filter functions of the listener's outer ears. These functions depend on the exact shape of the individual's ear so the filter functions were recorded from the ears of each subject. The recording method is described in detail elsewhere^59^ but is briefly summarised here. The subject was seated in the middle of an anechoic chamber and test signals (Golay codes) were played from a loudspeaker placed sequentially at each of 393 test locations equally spaced on an imaginary sphere surrounding the subject. The responses were recorded using miniature microphones (Sennheiser, type KE4-211-2) inserted into the subject's ear canals, filtered (200 Hz to 16 kHz) and digitised at 80 kHz (TDT System II). The location dependent component was extracted from each transfer function^60^ and used to filter the speech and masker stimuli before presentation over in-ear tube phones (Etymotic ER-2). Target talkers were rendered so that they appeared directly ahead of the listener. Maskers were either collocated with the target (Figure 1; collocated condition) or located 60° to the left of the midline on the audio-visual horizon (separated condition; Figure 1). All listening experiments were carried out in a sound attenuating audio-booth or the anechoic chamber.

The fidelity of the rendered VAS was tested for each subject by comparing the speech reception thresholds and the total spatial release from masking obtained using a free field presentation in the anechoic chamber with those obtained using a virtual space presentation over ear-phones. The target was a single female talker. Four subjects were tested using the speech matched noise maskers and four subjects using garbled speech maskers. Across the pool of 8 subjects there was very good correspondence between the absolute speech reception thresholds obtained in VAS and those obtained in the free field (mean difference in the SRTs in the collocated condition 0.2 dB, S.D. 0.9 dB; separated condition −0.3 dB S.D. 1.2 dB). Differences in the total spatial release between free field and VAS conditions were also very small (means −0.5 dB S.D. 0.8 dB). This indicates that the individualised virtual auditory space was a high fidelity rendering of the free field experience, at least as judged by performance on the task used in this experiment.

### Testing protocol

Experiments were conducted using lists of 35 individual test sentences; each test consisting of one randomly selected female target talker with the call sign “baron” and played concurrently with two randomly selected masker stimuli with combined spectral energy level matched to that of the single target at 0 dB SNR. In the each experiment there were three masker conditions (i) two different female talkers with call signs other than “baron”; (ii) speech-like maskers based on 2 other female talkers or (iii) noise matched in spectrum and overall envelope to 2 other female talkers. For each masker condition there were four listening conditions (i) stimuli collocated in VAS; (ii) stimuli separated in VAS; (iii) diotic presentation of the left ear signal in the collocated condition and (iv) diotic presentation of the left ear signal for the separated condition. The masker condition and listening condition were constant for any one list, but the different lists were presented in a randomized order.

Masker level remained constant (corresponding to 65 dB sensation level), while the level of the target talker varied randomly across seven levels evenly spaced between a specified upper and lower limit (5 repeats at the 7 levels in each list). For the first list with a particular masker and spatial configuration, the upper limit was set at 0 dB target to masker level (i.e. same level as the masker stimulus) and the lower limit at −45 dB (i.e. the target talker was 45 dB less than the combined masker stimulus). Each list was repeated 4 times in randomized order, with upper and lower limits adaptively varied so that if a subject scored 0% or 100% at a given level on a previous test, the new lower/upper limit was set 3 dB inside this level. The extreme ranges could also be varied to ensure SNRs resulting in 0% and 100% were also tested. This procedure ensured (i) robust fits of the psychometric functions and that a major proportion of the data was collected around the listener's 50% threshold so as to obtain a robust measure of threshold.

At the beginning of each list, two sample sentences were presented as preparatory stimuli, the results of which were not recorded. For these samples, the target talker level was set to 0 dB target to masker ratio while the other parameters matched those of the following 35 trials. A psychometric function was generated using a cumulative Gaussian using a maximum likelihood estimator^61^. The target to masker ratio corresponding to the 50% point on the psychometric curve was used as an estimate of the speech reception threshold (SRT). A bootstrapping procedure^62^,^63^ was used to resample each psychometric function 500 times. Standard deviations for SRTs were estimated by calculating the standard deviation of SRTs obtained from the re-sampled curves.

## Author Contributions

S.C. designed the experiment, C.C. collected the data, C.C. and S.C. performed the analysis and S.C. wrote the paper. The authors declare no conflict of interest.

## Figures and Tables

**Table 1:** Filter bands used in generating “garbled” speech

Band #	Frequency (Hz)	Band #	Frequency (Hz)	Band #	Frequency (Hz)
1	50–110	9	990–1240	17	5130–6220
2	110–180	10	1240–1500	18	6220–7520
3	180–260	11	1500–1940	19	7520–9100
4	160–360	12	1940–2330	20	9100–10990
5	360–470	13	2330–2850	21	10990–13260
6	470–620	14	2850–3470	22	13260–16000
7	620–720	15	3470–4230		
8	720–990	16	4230–5130		
